# Maturation of the Meniscal Collagen Structure Revealed by Polarization-Resolved and Directional Second Harmonic Generation Microscopy

**DOI:** 10.1038/s41598-019-54942-0

**Published:** 2019-12-05

**Authors:** Maxime Pinsard, Sheila Laverty, Hélène Richard, Julia Dubuc, Marie-Claire Schanne-Klein, François Légaré

**Affiliations:** 1 Institut National de la Recherche Scientifique, Centre Énergie Matériaux Télécommunications (INRS-EMT); 1650 Boul. Lionel-Boulet, Varennes (QC), J3X 1S2 Canada; 2 Comparative Orthopedic Research Laboratory, Department of Clinical Sciences, Faculté de médecine vétérinaire, Université de Montréal, Saint-Hyacinthe (QC), J2S 2M2 Canada; 3 Laboratoire d’Optique et Biosciences (LOB), École Polytechnique, CNRS, Inserm, Institut Polytechnique de Paris, F-91128 Palaiseau, France

**Keywords:** Microscopy, Imaging, Microscopy

## Abstract

We report Polarization-resolved Second Harmonic Generation (P-SHG) and directional SHG (forward and backward, F/B) measurements of equine foetal and adult collagen in meniscus, over large field-of-views using sample-scanning. Large differences of collagen structure and fibril orientation with maturation are revealed, validating the potential for this novel methodology to track such changes in meniscal structure. The foetal menisci had a non-organized and more random collagen fibrillar structure when compared with adult using P-SHG. For the latter, clusters of homogeneous fibril orientation (inter-fibrillar areas) were revealed, separated by thick fibers. F/B SHG showed numerous different features in adults notably, in thick fibers compared to interfibrillar areas, unlike foetal menisci that showed similar patterns for both directions. This work confirms previous studies and improves the understanding of meniscal collagen structure and its maturation, and makes F/B and P-SHG good candidates for future studies aiming at revealing structural modifications to meniscus due to pathologies.

## Introduction

The meniscus is a semilunar fibrocartilaginous structure interposed between the femoral condyle and the tibial plateau in the knee joint. The meniscus is essential for load transmission across the articular surfaces, for femorotibial joint stability and for long-term joint health^1^. Degradation of the meniscal tissue can increase articular cartilage strain^2^, and may lead to cartilage degeneration and osteoarthritis^3^. Knowledge of the complex structure of the meniscal extracellular matrix (ECM) has increased thanks to emerging technologies for *in situ* imaging of intact specimens, such as Optical Projection Tomography (OPT)^4^. In particular the arrangement of meniscal fascicles^4^, its tie-fiber organization^5^, and the menisco-tibial ligament insertion transition have all recently been revealed by investigation of bovine samples^6^.

SHG microscopy is a recent and powerful technique to image the structure of biological specimens as it provides submicron spatial resolution, has low phototoxicity and a high depth selectivity and penetration. In this respect, SHG imaging is similar to multiphoton-excited fluorescence microscopy^7^. However, important differences exist: it is a coherent process sensitive to the phase-matching conditions where the measured signal arises from constructive/destructive interferences, it is also instantaneous and free from photobleaching as the signal conversion is due to a structural arrangement and does not involve electronic transition^8^. SHG microscopy has been used to image fibrillar collagen in specimens including type II collagen in articular cartilage^9–16^. Furthermore, because of its coherent nature, the detection of the signal in the direction of propagation (forward - F) provides different imaging features compared to the backward (B) direction^17^. The F/B ratio increases with the level of homogeneity of noncentrosymmetric structures within the focal volume and has been related to the size of the collagen fibrils for collagen rich tissues^18,19^.

The complex noncentrosymmetric structural organization of meniscal collagen has been previously imaged using Second Harmonic Generation (SHG) microscopy^5,20–23^. SHG microscopy has revealed a variety of meniscal structural features including: the fiber bundle organization^24^, the arborization of the tie-fibers^5^ and the interaction of primary cilia with the ECM^25^ in healthy menisci. The effect of degradation on ECM density and cell accumulation^26^, of cyclic loading^27^ and the meniscal integrity following repair^20^ have also been imaged by SHG microscopy. Furthermore large images of the whole joint (cartilage and meniscus) were imaged by multiphoton fluorescence and SHG^28^, and even *in-vivo* and minimally processed joints using SHG microendoscopy^29^. SHG and Coherent Anti-Stokes Raman Scattering (CARS) microscopy have also been employed to characterize artificial meniscus-like implants^30^. However, up to now, the local orientation of the meniscal collagen structures has been mainly characterized by OPT, only to a resolution of ~15 µm permitting identification of the collagen fascicles but not of the individual fibers^4^. These fibers were later imaged by SHG microscopy in some areas from bovine samples^5^, but their orientation was discussed only in terms of the SHG intensity. The latter is subject to interference due to the coherent nature of the process, that can result in masking the real underlying structure^31^ therefore preventing imaging of the fibril orientation and nonlinear tensor properties on its own^32^. Polarization-resolved SHG (P-SHG) was developed to override this limitation, and has been shown to accurately reveal the alignment distribution of collagen fibrils in various tissues such as tail tendon or skin^32–34^, but has never been reported in meniscus.

The influence of age on the mechanical response of meniscal connective tissue has been studied at the nanoscale in human menisci using atomic force microscopy^35^. The propagation of collagen II deposition has been investigated in the mouse (using an anti-collagen II bioclone) from the inner to outer border, during meniscal growth from foetus to adult^36^. Furthermore the relative area of meniscus versus articular cartilage has also been characterized during human foetal gestation^37^. In addition, the meniscus has been shown to increasingly develop cartilaginous properties during maturation^38^. A recent study also compared vertical meniscal sections in bovine joints from foetuses and adults^39^, and reported that the spacing between circumferential bundles of fibrils is much higher in foetuses than in adults.

Here, we demonstrate the unique potential of SHG and P-SHG to measure the collagen structure in the meniscus, and we use these two techniques together with histology to characterize the structural differences between equine foetal and adult meniscal ECM. Understanding and imaging the meniscal ECM could provide new knowledge to support studies on meniscal degradation events in joint degeneration, and potentially new insights towards meniscal regeneration in the future.

## Results

### Forward and backward SHG

**Figure 1:**
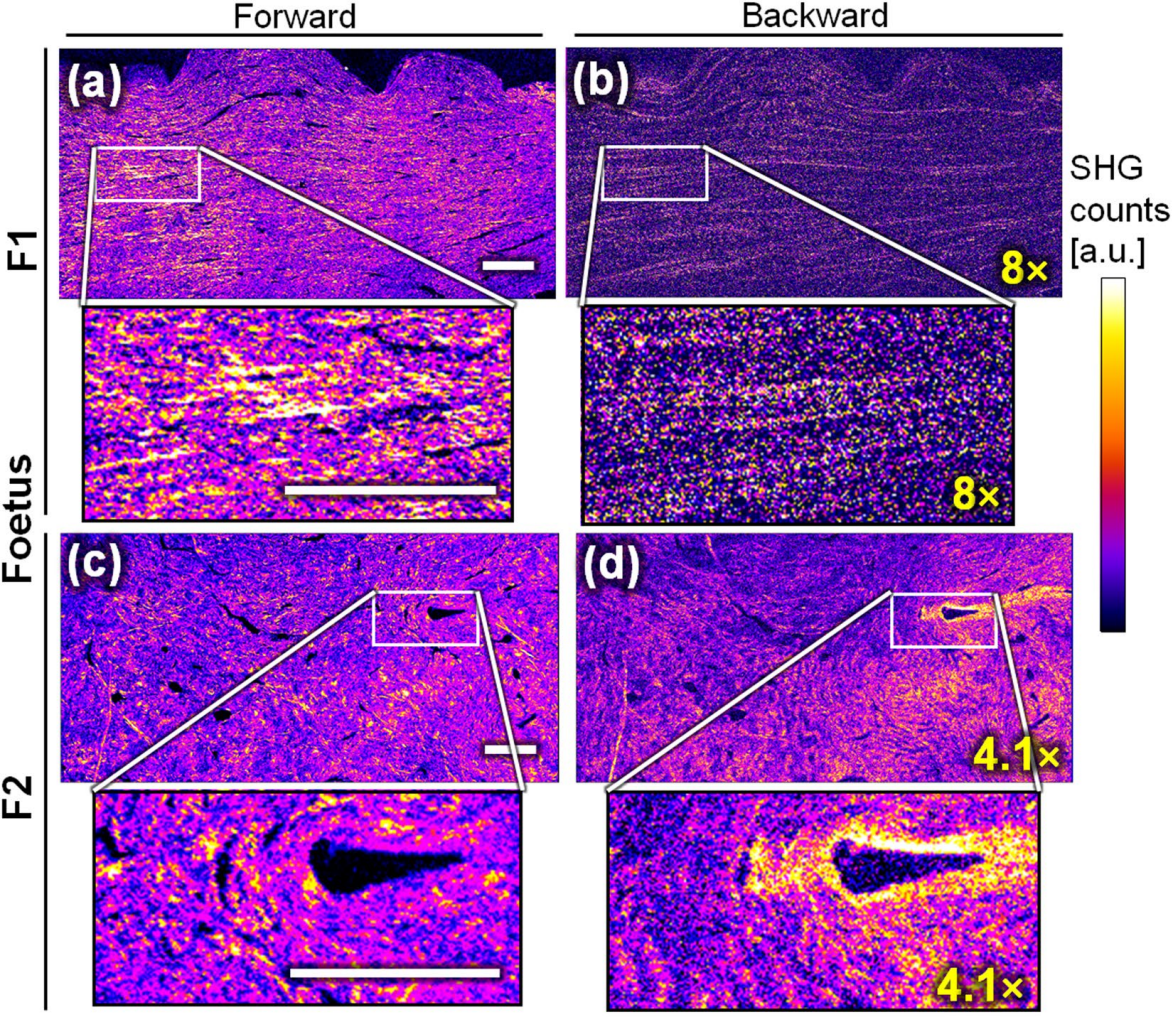
Forward SHG (left, (**a,c**)) and backward SHG (right, (**b,d**)) from two menisci samples from equine foetuses ((**a,b**), F1 and (**c,d**) F2) knee joints). F1 ((**a,b**)) shows a tissue that is mostly homogeneous (horizontal patterns), whereas F2 ((**c,d**)) shows more randomly oriented patterns. Both menisci show similar images in forward and backward directions. The images of the same samples (**a,b or c,d**) are displayed using the same look-up table, but the backward images have been multiplied by a factor indicated in yellow because less signal is physically detected in this direction compared to forward. Scale-bars: 200 μm.

**Figure 2:**
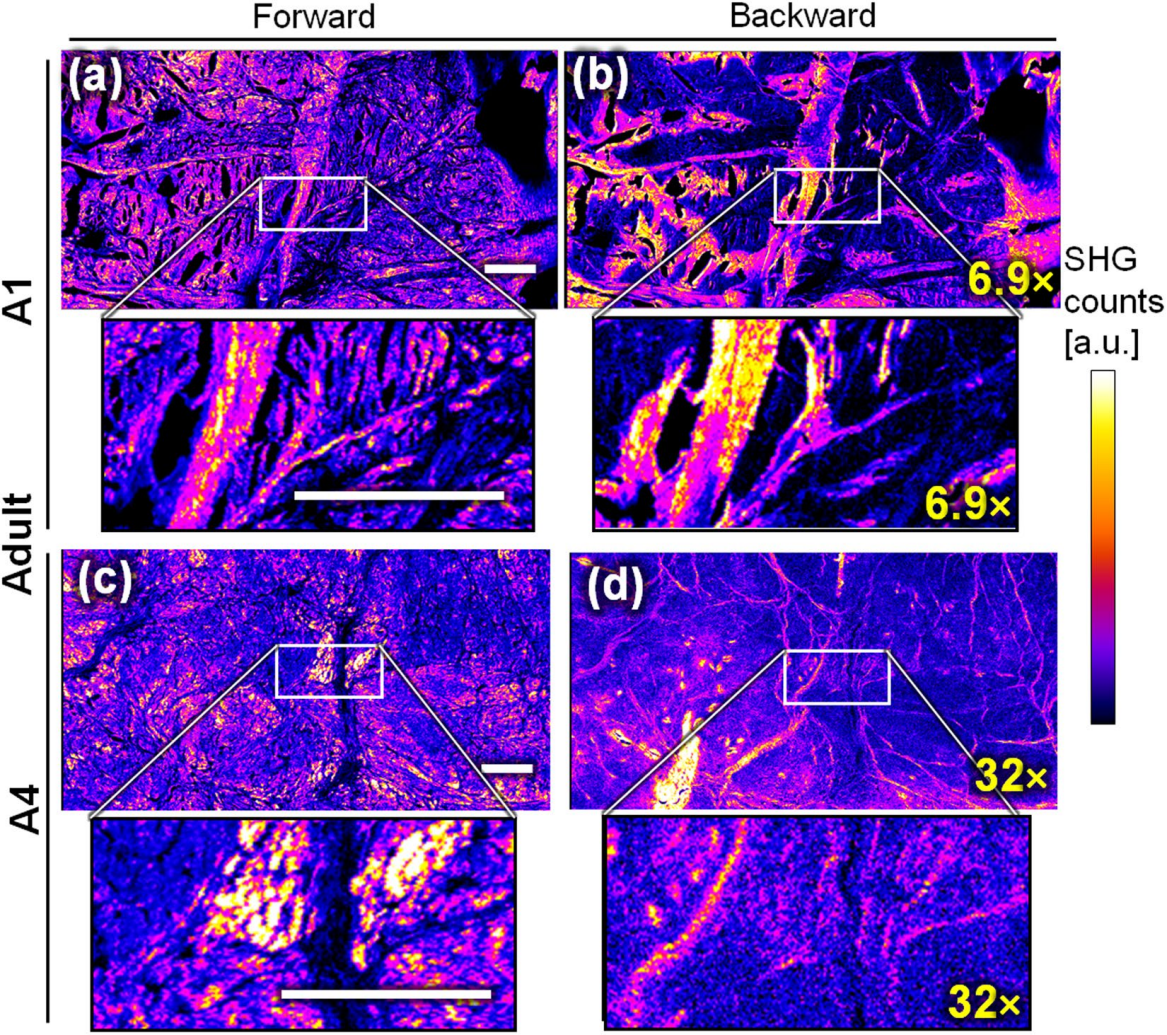
Forward SHG (left, (**a,c**)) and backward SHG (right, (**b,d**)) from two menisci of equine adults (A1 for (**a,b**) and A4 for (**c,d**)) knee joints. Both menisci show different patterns in forward and backward directions: the backward images better highlights the thick structures, mostly identified as fibers. The images of the same samples (**a,b or c,d**) are displayed using the same look-up table, but the backward images have been multiplied by a factor indicated in yellow because less signal is physically detected in this direction compared to forward. Scale-bars: 200 μm.

Representative images of the ECM from the central body of two foetal and adult menisci visualized by standard SHG (using circular polarization) are presented in Figs. 1 and 2 respectively. Only collagen fibrils are visible as they are the only non-centrosymmetric material^5^. Additional images of all foetal and adult menisci can be found in the Supplementary information (respectively Figs. S3 and S4).

The SHG images of Fig. 1 reveal that the collagen of foetal menisci shows similar patterns in both forward and backward SHG directions. Figure 1 shows that F1 (a & b) has aligned horizontal fibrils over the whole region-of-interest (ROI), and that F2 presents numerous different orientations in the ROI (c & d, see also Table S1). For both samples, the visible patterns are similar, and forward and backward SHG images present equivalent features. This is particularly visible on the overlay of the forward and backward channels of Figure S2 (a&b, Supplementary information).

In the case of mature specimens (adults, Fig. 2), the forward images present numerous differences from the backward ones. In the backward direction, thick collagen fibers are visible while the signal between the thick fibers is strongly reduced, or with zero signal (see also Figure S2 c&d). It is worth noting that, here, the retro-reflection of the forward SHG on the backward image seems to be negligible: no pattern from the forward images (a & c) is visible on the backward image (b & d). Thus, the experimental precaution indicated for instance in Légaré *et al*.^40^ was not here necessary to obtain a proper backward image.

### Polarization-resolved SHG (P-SHG)

**Figure 3:**
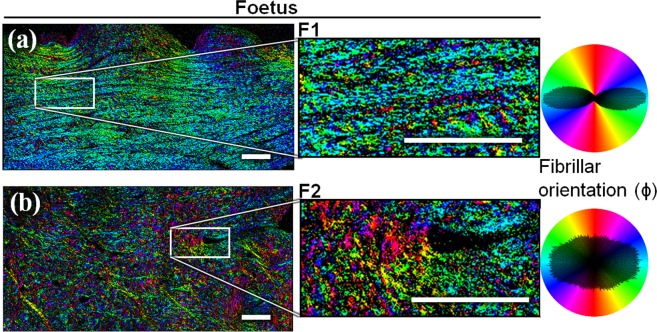
Collagen fibril orientation in the imaging plane (ϕ) measured by P-SHG, for the same foetus specimens (F1 (**a**) and F2 (**b**)) as in Fig. 1. The foetus F1 (**a**) has a homogeneous orientation: the polar histogram reveals two directional lobes. The foetus F2 (**b**) exhibits a random orientation, almost equally scattered over all the angles: the polar histogram is then a low-eccentricity ellipse. Scale-bars: 200 μm.

**Figure 4:**
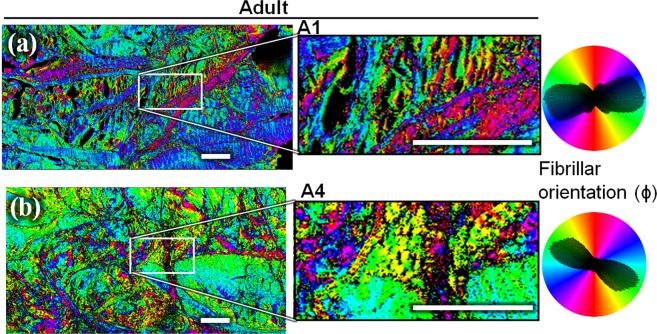
Collagen fibrils orientation in the imaging plane (ϕ) measured by P-SHG, for the same two adults’ specimen (A1 (**a**) and A4 (**b**)) as in Fig. 2. These mature menisci have an organized structure, with some thick fibers that clearly delimitate bundles of homogeneous fibrils. On the polar histograms, the orientation shows a principal component coupled with some other components corresponding to the numerous – but still limited – orientations. Scale-bars: 200 μm.

Collagen fibril orientation in the imaging plane obtained by P-SHG imaging is shown in Fig. 3 for the foetal menisci (exact same zones as in Fig. 1), and in Fig. 4 for the adult ones (exact same zones as in Fig. 2). Images of other foetus and adults can be found in the Supplementary information, Fig. S5 (foetus) and S6 (adult). The foetal meniscal fibrils are either homogeneous in orientation (Fig. 3(a)), or random (Fig. 3(b)), whereas all the adult samples show clusters of homogeneous orientations. On the polar histogram (revealing the overall orientation of the collagen fibrils), the random distribution is seen by an almost circular pattern, whereas an homogeneous distribution leads to a directional double-lobe. These P-SHG images confirm what was shown in the backward SHG images (Figs. 1 and 2): in the adult meniscus (Fig. 4(a),(b)), a complex network arrangement of thick fibers interlaces a rather homogeneous assembly of orthogonal fibril bundles.

### Quantitative comparison

**Figure 5:**
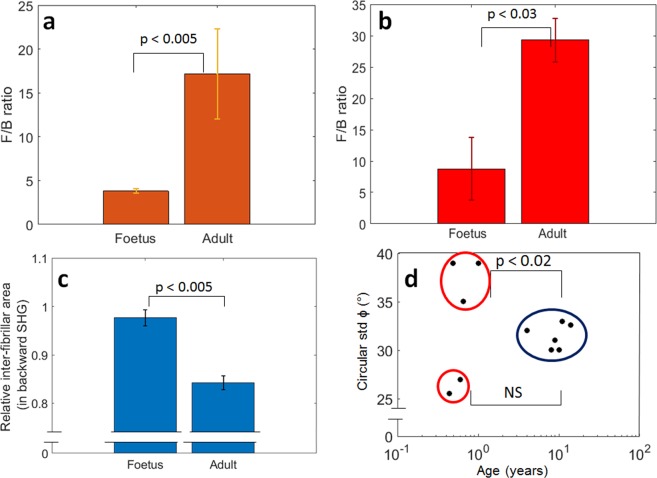
Quantitative results of F/B and P-SHG. Forward over backward ratio (F/B) over the whole image (**a**) or for the inter-fibrillar area (**b**) for all the foetus and adult menisci. (**c**) Relative inter-fibrillar area measured in backward SHG, i.e. the percentage of inter-fibrillar area compared to the whole image. (**d**) Circular standard deviation (std) of the P-SHG fibril orientation in the image plane (ϕ): the adult (in dark blue) have values around 32°, whereas the foetuses (in red) split in two groups: one having a value of ϕ circular std rather low (~26°), the other one a larger value (~37°). In each graph, the mean of the different values was taken, and the error bars indicate the standard error of the mean (SEM) of the serie. The statistical significance (NS: non-significant, p < 0.05: significant) was tested using the non-parametric Wilcoxon-Mann-Whitney test. The set of samples consist of n = 5 for foetuses and n = 6 for adults, which gives an acceptable statistical representation given the difficulty to obtain foetus samples.

The pixel-wise ratio of forward over backward SHG signals (F/B) was averaged first over the whole image in Fig. 5(a), and then by separating the thick fibers from the residual tissue (inter-fibrillar area, see Methods) in Fig. 5(b). The F/B SHG for the thick fibers area are similar for adult vs foetus specimens (not shown), while the remaining tissue (i.e. inter-fibrillar area) shows an F/B around 4.5 for the foetus and around 28 for the adult: this is consistent with what is observed in Figs. 1 and 2. The F/B of the whole image is also significantly different for the adult versus the foetus. In addition, the difference observed in the structure of SHG images was assessed by the ratio of non-fibrous over fibrous areas: as seen in Fig. 5(c), the area without those thick fibers (inter-fibrillar area) is close to 98% for the foetuses whereas it is around 90% for the adult, with a significant statistical difference between both values.

Moreover, the differences between the fibril orientation distributions observed in Figs. 3 and 4 were verified by computing the circular standard deviation (std) of the angle ϕ, for every meniscus imaged in P-SHG. The results are reported in Fig. 5(d). The foetuses are clearly split into two groups, as the difference between the homogeneous and the random fibril orientation is too high to merge them together. Noteworthily, this discrepancy cannot be explained by the age of the foetuses (see Table S1 in the Supplementary information) nor the type of menisci (lateral versus medial). It could rather be partly due to the relatively small site studied, and the difficulty of having all measures at the exact same site in each samples. The two foetal samples with a homogeneous orientation (F1 and F3, see Table S1) do not show a significant difference from the adult group (too few samples), but the group of foetuses having random fibril orientation does show a significant difference. These statistics suggest that some subgroup of foetal samples exhibit different fibril orientation compared to the adults, while the reason to have different subgroups in the foetal samples remains unclear.

## Discussion

Collagen, which plays a central role in the architecture of the tissue^41^, is the principal component of the adult meniscal ECM (70%), of which 60% is type II and 40% type I^42^. The ordering of the collagen fibrils within the focal volume of excitation (i.e. at the micron-scale) is usually probed by P-SHG using the anisotropy parameter ρ^43^ (see Methods section for definition), but this method has been mainly applied to tissues composed of only one type of collagen. Indeed, despite having similar sequences of amino-acids^44^, collagen I and II have different properties: they are biochemically different, and their fibrils have different sizes^12^. It is thus a challenge to measure structural parameters such as ρ in such tissues. In previous studies of cartilage tissues, Romijn *et al*.^44^ have differentiated collagen I and II but only at some specific pixels, resulting in a ρ mapping with a relatively high level of uncertainty^44^. This discrimination is also challenging because the average ρ in collagen I and II was found to be similar^44^.

**Figure 6:**
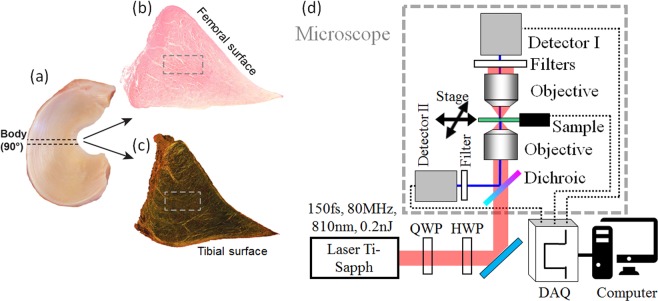
Study design. The meniscus was removed from the equine knee femorotibial stifle (joint) and its body was sectioned at 90° (**a**). The triangular-shaped coronal (circumferential) sections were deposited on a microscope slide. (**b**) HEPS stain for standard histology and (**c**) Picro-sirius red stain for collagen. The rectangle in (**b**) shows the central area of the tissue investigated in SHG and P-SHG microscopy. (**d**) Schematic view of the set-up used for P-SHG and F/B SHG. A femtosecond laser directly excites the sample without being scanned, the image being done by moving the sample laterally with a motorized stage, synchronized to the acquisition by a multifunction card (DAQ). QWP: Quarter-wave plate, HWP: Half-wave plate. In P-SHG, the dichroic mirror can be removed to ensure a better polarization control, as the signal is captured in the forward direction. For F/B measurements, a dichroic filter is inserted before the excitation objective to send the backward SHG to a 2^nd^ detector (II).

Moreover, the semilunar and concave form of the meniscus (see Fig. 6 in the Methods), and the 3D arrangement of its collagen results in fibrils pointing out of the image plane: this highly complex structural organization artificially modifies the measured ρ^44^. As a result, the ρ values averaged over the whole images were undistinguishable between our samples of foetal and adult menisci (not shown here), despite clear different features observable in the images. Overall, the shape and contrast of the polarimetric diagrams extracted at each pixel cannot be exploited due to these complex effects. This comes at a high cost since this is usually the main feature exploited in P-SHG. For instance, this precludes the classification of these tissues by the method previously described by Rouède *et al*.^41^, that mainly relies on ρ.

Despite this limitation, P-SHG can be used to measure the in-plane fibrillar orientation ϕ, as it exploits only the average orientation of the polarimetric diagram. The ϕ maps with or without the Kleinman filter condition were similar (data not shown), which shows that these maps are not sensitive to the precise value of the susceptibility tensor components, and that P-SHG’s ϕ provides a robust way to measure these orientation maps. The ϕ-map in P-SHG (Figs. 3 and 4) probes the orientation of collagen fibrils at a local scale (pixel resolution, i.e. few μm), while the circular std (see methods section) of Fig. 5(d) measures the global tendency of alignment (at the field-of-view scale, i.e. few mm). A circular std has been chosen because it is a good metric of the dispersion of the ϕ distribution. The collagen fibrillar orientation of the adults (discernible in Fig. 2 and clearly revealed in Fig. 4) seems to confirm what was suggested in Andrews *et al*.^5^: some clusters of fibrils exhibit homogeneous orientation, and are separated by thick fibers. We interpret the clusters as the fascicles and the thick fibers as some parts of the tie-fiber sheets that are described in Andrews *et al*.^5^. The low SHG signal of the inter-fibrillar area observed in backward images is consistent with the fact that the fascicles are perpendicular to the thick fibers in the image plane^5^. Furthermore, the F/B ratio for these zones is higher in average, consistently with what is expected for out-of-plane fibrils^22^.

The study of the forward and backward SHG (F/B) provides an analysis on the directionality of the SHG signal. The forward SHG image shows at each pixel the degree of phase-matching, with a selectivity on the ordered structures (within the focal volume), at the size of the SHG wavelength λ_SHG_. The backward SHG reveals smaller or more random structures in a complementary way^17^. The F/B ratio is then smaller for the more disorganized structures - or for structures organized on a size smaller than λ_SHG_^17^ - and increases with the fibril diameter, or with the diameter of bundle of fibrils of same polarity^18,45^. This ratio is averaged over the whole field-of-view and thus provides global information on the collagen bundles in terms of size and arangment. Specifically, it is expected to be smaller for type II collagen, which is known to form fibrils with smaller diameters (few tens of nm^46^) than type I collagen (few hundreds of nm)^47,48^. Collagen II is also present in low amounts in foetuses, but increases with age^36,38^. In addition, tie-fibers in mature samples have higher quantities of it compared to the other regions like fascicles^49^. We therefore expect that the tie-fibers exhibit a lower F/B ratio than the rest due to the type II collagen fibrils present in this structure: accordingly, we have observed that the tie-fibers were the main visible patterns in the backward direction. However, the adults that contains more tie-fibers than foetuses would have a smaller global F/B ratio. This confirms that F/B SHG cannot unambiguously discriminate between type I and type II collagen or measure their respective quantities. Nevertheless, it demonstrates that it is a useful structural probe – complementary to histological assessment – sensitive to the different spatial organizations of the collagen (type II or mix type I/II) that varies with age.

Interestingly, the result of Fig. 5(c) confirms what was measured by Qu *et al*.^39^ with multimodal multiphoton microscopy (SHG and two-photon fluorescence), using a local thickness measurement: the inter-fibrillar area reduces with maturity in meniscus. However, it is more precise to use P-SHG to circumvent interference pattern artifacts, as mentioned in the introduction. It is also noteworthy that the change from a random collagen meshwork to a more organized arrangement with maturation - that we have observed here in meniscus - has also been demonstrated in developing cartilage^50^. Additionally, cartilage maturation has been shown to be coupled with the apparition of different zones^50,51^, which is consistent with the formation of clusters of fibrils in meniscus that we have observed in this study.

To conclude, we report, for the first time to our knowledge, the mapping of the in-plane orientation of the collagen fibrils within the meniscal tissues over large ROIs. The observed structure of the collagen network in adult samples is in agreement with what has been previously reported (with other techniques) in bovine menisci^4,5^: relatively thick tie-fibers oriented radially, interlacing some fascicles oriented circumferentially. Moreover, this organized structure is not identified in the foetal meniscus: the latter is composed of either a random fibril organization, or no network at all with only parallel fibrils. This study confirms the importance of SHG microscopy, especially the F/B as a structural probe for the study of the meniscal – and potentially the cartilage’s – ECM, as well as the capacity of P-SHG to map the fibril orientation even with a mix of different collagen types. Both techniques are shown to be automated and applicable within a reasonable acquisition time, enabling large scale studies in the future. They also provide quantitative results which can be significant for further studies of the structure of meniscus at the micron-scale. We anticipate their future use for characterization of degeneration in the meniscus or cartilage, and overall furthering the understanding of such tissues.

## Methods

### Sample preparation

This project was approved by the Institutional Animal Care and Use Committee (IACUC) of the Faculté de médecine vétérinaire, Université de Montréal. Tissues were obtained from a local slaughterhouse and from horses donated by their owners for research. The age of equine foetuses (n = 5) was estimated using crown-rump measurements as previously described^3,52,53^. Adult samples (n = 6) were also studied, and had been banked from a previous study. The femorotibial joints were freed of soft tissue, inspected and the menisci harvested. Menisci were included in the study if the macroscopic appearance of all surfaces was normal (no fibrillations or tears) and if the joint of origin was also macroscopically intact (no cartilage erosions or evident pathology). Sample listing can be found on Table S1 of Supplementary information. Each meniscus was then laid over a protractor with the femoral surface uppermost and the cranial border aligned with the angle 0 as described previously^3^ (see Fig. 6(b),(c)). A slice orthogonal to the circumferential direction^54^, i.e. in the vertical-radial plane^5^ was cut in the body of the meniscus. The menisci were placed in 10% formaldehyde for 2 h and then transferred to Ethylenediaminetetraacetic acid (EDTA, to avoid calcification effects on cutting) 20% for 2 weeks prior to paraffin embedding and subsequent sectioning. Five micron sections were cut (Fig. 6(a)) and then placed on a microscope slide (1 mm thick) covered by a thin coverslip (#1.5 H, Thorlabs). The central part of this triangular-shaped tissue (see Fig. 6(b),(c)) was imaged on a microscopy slide oriented upside-down, with the coverslip facing the incoming light since the microscope objective is made to image through a thin coverslip (see Fig. 6(d)). This segment (body) of the meniscus was selected because it is denser in collagen than in proteoglycan, contrary to other sites^1^.

Five micron sections were also cut and stained with haematoxylin, eosin, phloxine and saffron (HEPS) as well as Picro-sirius red to illustrate the collagen network architecture under polarized light microscopy (see Supplementary information Figs. S1 and S2). All slides were digitalized with a LeicaDM 4000B microscope and Panoptiq v.1.4.3 computer software. All the images of the ROI of the central part are presented with the same geometry as in Fig. 6(b), with the tibial surface at bottom and the inner border to the right.

### SHG microscopy

A mode-locked Ti:Sapph oscillator (Tsunami, Spectra Physics) delivering ~150 fs pulses at 810 nm and 80 MHz rate was used as the laser source. The microscope is a modified commercial laser-scanning setup (iMic, Thermo-Fisher Scientific Munich GmbH), where a plane mirror was inserted before the objective to by-pass the laser-scanning, and send the beam directly to the objective to perform sample-scanning with a translation stage (MLS203, Thorlabs). An achromatic telescope was used to re-size the beam to overfill the back aperture of the objective (UplanSApo 20 × , strain-free for good polarization control, NA 0.75, air immersion, Olympus). The polarization was controlled by a multi-wavelength half-wave plate (HWP, at 400 and 800 nm), and by an achromatic quarter-wave plate (QWP, 700–2500 nm, B. Halle) placed before the microscope (see Fig. 6(d)). A mechanical motor was used to vertically move the objective to find the proper focus position. Signals were collected in the forward direction using an objective with a numerical aperture of 0.75 (same reference as the excitation one) and detected on a photomultiplier tube (PMT - R6357 amplified with a C7319 unit, Hamamatsu Photonics, Japan) set at 900 V using appropriate spectral filters (two FF01-720/SP-25 and a FF01-405/10, SEMRock, Rochester, NY, USA). Scanning and signal acquisition were synchronized using a custom-written Python (www.python.org) software and a 6110 multichannel I/O acquisition card (National Instruments). Images were recorded using 50 µs pixel dwell-time. The sample-scanning allowed to do rather large scans (2000 × 1000 μm, 2 μm/pixel) in just one acquisition (no mosaic reconstruction) and in a reasonable acquisition time (55 sec), providing that the scan parameters are optimized and the synchronization between the motor and the acquisition DAQ card is controlled. Each image was acquired 3 times to perform an average measurement. These acquisition parameters were the same for the two techniques F/B SHG and P-SHG. The average power on the sample was adjusted to 20 mW, corresponding to roughly 0.3 nJ/pulse. Raw data visualization was performed with FIJI-ImageJ (NIH^55^) and image processing was performed with MatLab (The MathWorks). All the images are presented with the full imaged areas, and oriented with the femoral surface on top and the outer surface on the left (as in Fig. 6(c)).

### Forward and backward SHG measurements (F/B SHG)

A circular polarization of excitation was used to image all structures independently of their orientation in the image plane. A long pass dichroic mirror (FF735-Di01-25 × 36, SEMRock) placed at the back-focal plane of the objective was used to reflect the SHG generated in the backward direction (epi). This dichroic mirror is translatable, and was inserted only when backward imaging was performed (using circular polarization), not for linearly polarized excitation. To calibrate the F/B measurements i.e. to take into account that the detection efficiency is different in forward and backward directions, we used the isotropic emission of the two-photon excited fluorescence signal from Coumarin 440 (diluted in ethanol), because its fluorescence (400–460 nm) lies partly in the range of the SHG filters (405 ± 5 nm). The obtained ratio between forward and backward directions was 0.4. The F/B ratio was calculated by an established method^17^ by taking the median of the pixel-wise F/B signal in the whole field-of-view, the forward and backward images being the average of the 3 acquired frames.

### Polarization-resolved SHG (P-SHG)

A perfect linear polarization is needed for P-SHG, so the polarization was calibrated with the routine developed in Romijn *et al*.^56^, using a modified version of their MatLab code^57^. This enables to use a varying linear polarization (from 0 to 180°) that stays linear even if the waveplates are placed before the input of the commercial microscope. It is worth noting that the dichroic used in F/B SHG measurements was removed for P-SHG, and that sample-scanning avoids any other source of polarization distortion compared to the standard laser-scanning. As we performed the SHG measurements, images were acquired for 18 polarization states, spaced by steps of 10° so that the full range of [0, 170°] was covered (the rest of the polarizations being redundant).

### Theory and data treatment in P-SHG

For a complete description of P-SHG, see Teulon *et al*.^58^. The collagen fibrils are here assumed to have cylindrical symmetry (C_∞_ group), and we neglect the chiral components of their nonlinear susceptibility tensor χ^(2)^. Furthermore, in the case where the fibrils are distributed around the imaging plane (which is reasonable for cartilage-like tissue), the out-of-plane orientation δ should be considered (δ = 0 meaning in the plane)^44^. Even if the Kleinman symmetry should here apply as NIR wavelengths are used to drive the SHG process, thus having energies far from any transition^44^, this condition is not assumed a priori to be able to verify it in data treatment. The independent non-zero components of the χ^(2)^ are then χ_33_, χ_31_ and χ_15_. The X axis in the XYZ frame corresponds to the same direction as the x axis in the xyz frame. The simplified expression for the two components of the SHG intensity (X being the direction of propagation) can then be written as^44^:

1 $$\begin{array}{c}{{{\rm{I}}}_{{\rm{Z}}}}^{2\omega }\propto {|{\chi }_{15}\cos \delta [{{\rm{A}}{\rm{s}}{\rm{i}}{\rm{n}}}^{2}(\theta -\varphi )+{{\rm{B}}{\rm{c}}{\rm{o}}{\rm{s}}}^{2}(\theta -\varphi )]|}^{2}\\ {{{\rm{I}}}_{{\rm{Y}}}}^{2\omega }\propto {|{\chi }_{15}{\rm{A}}\cos \delta \sin (\theta -\varphi )\cos (\theta -\varphi )|}^{2}\end{array}$$

where θ is the angle of the imposed polarization with respect to the Z-axis of the laboratory frame, and ϕ the orientation of the collagen fibril with respect to this axis. Also, *A* = *χ*_31_/*χ*_15_ and *B* = *χ*_33_/*χ*_15_cos^2^*δ* + (2 + *χ*_31_/*χ*_15_)sin^2^*δ*^44^, so that ${\rho }_{0}=B(\delta =0)/A={\chi }_{zzz}^{(2)}/{\chi }_{zyy}^{(2)}={\chi }_{33}/{\chi }_{31}$ is the anisotropy parameter (z being the fibril axis and y the axis orthogonal to X and z). The more standard calculation would assume δ = 0, A = 1 and B = *ρ*_0_, i.e. that the Kleinman condition applies and that the collagen lies in the imaging plane. Here, we process the P-SHG data with or without a filter that imposes abs(A) < 1.1, as in Romijn *et al*.^44^ (called the Kleinman filter from now on). We still expect to be close to the Kleinman symmetry (i.e. A ≈ 1) as already mentioned. ρ is in this case the anisotropy parameter in the frame of the tilted fibril, which can be written as *ρ* = *ρ*_0_cos^2^*δ* + 3sin^2^*δ* where *ρ*_0_ is the anisotropy parameter for no tilt^58^. Summing the above intensities and developing the sine and cosine gives^58^:

2 $${{\rm{I}}}_{{\rm{t}}{\rm{o}}{\rm{t}}}^{2\omega }={{\rm{a}}}_{0}+{{\rm{a}}}_{2}\,\cos \,2(\theta -\varphi )+{{\rm{a}}}_{4}\,\cos \,4(\theta -\varphi )$$

With

3 $$\begin{array}{rcl}{{\rm{a}}}_{0} & = & {\rm{K}}/2[1+{(({\rm{\rho }}-1)/2)}^{2}+2{(({\rm{\rho }}+1)/2)}^{2}]\\ {{\rm{a}}}_{2} & = & {\rm{K}}({{\rm{\rho }}}^{2}-1)/2)\\ {{\rm{a}}}_{4} & = & {\rm{K}}/8({\rm{\rho }}-3)({\rm{\rho }}+1)\end{array}$$

and K a constant that gathers different physical parameters. The exact same expression is obtained for the standard case of δ = 0 by substituting ρ_0_ to ρ^43^. A custom MatLab routine was then used to extract the relevant information from the P-SHG images. Briefly, a spatial FFT algorithm with respect to the angle θ is used to compute the Fourier transform (variable Ω) of the measured intensity^59^:

4 $${{\rm{I}}}_{{\rm{F}}{\rm{F}}{\rm{T}}}(\Omega )={\alpha }_{0}{\rm{D}}(0)+{\alpha }_{2}{{\rm{e}}}^{2{\rm{i}}{\varphi }_{2}}{\rm{D}}(2-\Omega )+{\alpha }_{4}{{\rm{e}}}^{4{\rm{i}}{\varphi }_{4}}{\rm{D}}(4-\Omega )+{\rm{c}}.{\rm{c}}.$$

where c.c. is the conjugated complex, and D the Dirac function (Fourier-Transform of cos θ).

The estimation ϕ_2_ (resp. ϕ_4_) of the relative orientation ϕ could then be extracted from the first exponential: $\Omega$ = 2 (resp. from the second exponential: Ω = 4): ${\rm{n}}{\varphi }_{{\rm{n}}}={\rm{A}}{\rm{r}}{\rm{g}}[{\alpha }_{{\rm{n}}}{{\rm{e}}}^{{\rm{i}}{\rm{n}}{\varphi }_{{\rm{n}}}}]$ (n = 2 or 4). Then, ${\varphi }_{2}=0.5{\rm{a}}{\rm{t}}{\rm{a}}{\rm{n}}2({\alpha }_{2}{{\rm{e}}}^{2{\rm{i}}{\varphi }_{2}})$ is directly in [−π/2, π/2] because the 2-arguments arctangent function atan2 casts its result in]−π, π]. For ϕ_4_, a simple arctangent must be used to avoid a wrong re-casting^59^, *ϕ*_4_ which is then in [−π/8, π/8]. Putting ${\rm{\beta }}={{\rm{\alpha }}}_{2}^{2}/({{\rm{\alpha }}}_{2}^{2}+4{{\rm{\alpha }}}_{4}^{2})$, it has been shown that the combination ϕ = βϕ_2_ + (1 − β)(ϕ_4_+mπ/4) gives the most accurate result^59^, with m in {−2, −1, 0, 1, 2} calculated to minimize the quantity $|{\varphi }_{2}-{\varphi }_{4}|$^59^.

### Polar histogram for P-SHG

The values of orientation ϕ obtained in P-SHG are circular, so their histograms are better represented on polar plots. The orientation is measured, but the polarity remains unknown so the values of ϕ are obtained in [−π/2, π/2]. To plot the histogram in a full circle, the values of ϕ were thus duplicated by a central symmetry (which makes the polar histograms inherently centrosymmetric). The orientations are color-coded, as indicated on the histograms (e.g. red for vertical and cyan for horizontal orientations). The standard deviation (std) for the values of ϕ must also use a circular calculation, otherwise the boundaries of the distribution are not considered equal and continuous, as an angle should be^60^.

### Quantitative distributions

The Shapiro-Wilk’s test was first used to reject the normality of the distributions, and the difference was then tested using the Wilcoxon-Mann-Whitney test. The significance threshold was set to 0.05. Meniscus shows regions of low SHG signal meaning discontinuities of the visible collagen structure, attributed to either out-of-plane fibrils or zones with no collagen. The relative surface of these inter-fibrillar areas can be calculated in ImageJ: a threshold (ImageJ “Yen white”) was first applied to the backward SHG images to discriminate the fibrillated areas from the rest (see the masks in Fig. S7, Supplementary information). Then, the relative area in this binary image was easily calculated.

## Supplementary information

Supplementary information

## Data Availability

The datasets generated and/or analyzed during the current study are available from the corresponding author on reasonable request.

## References

[1] Andrews SHJ, Adesida AB, Abusara Z, Shrive NG (2017). Current concepts on structure–function relationships in the menisci. Connect. Tissue Res..

[2] Carter TE (2015). *In vivo* cartilage strain increases following medial meniscal tear and correlates with synovial fluid matrix metalloproteinase activity. J. Biomech..

[3] Dubuc J, Girard C, Richard H, De Lasalle J, Laverty S (2018). Equine meniscal degeneration is associated with medial femorotibial osteoarthritis. Equine Vet. J..

[4] Andrews SHJ, Ronsky JL, Rattner JB, Shrive NG, Jamniczky HA (2013). An evaluation of meniscal collagenous structure using optical projection tomography. BMC Med. Imaging.

[5] Andrews SHJ (2014). Tie-fibre structure and organization in the knee menisci. J. Anat..

[6] Andrews SHJ, Rattner JB, Jamniczky HA, Shrive NG, Adesida AB (2015). The structural and compositional transition of the meniscal roots into the fibrocartilage of the menisci. J. Anat..

[7] Denk W, Strickler JH, Webb W (1990). Two-photon laser scanning fluorescence microscopy. Science.

[8] Brown EB (2003). Dynamic imaging of collagen and its modulation in tumors *in vivo* using second-harmonic generation. Nat. Med..

[9] Mansfield J (2009). The elastin network: Its relationship with collagen and cells in articular cartilage as visualized by multiphoton microscopy. J. Anat..

[10] Brockbank KGM (2008). Quantitative second harmonic generation imaging of cartilage damage. Cell Tissue Bank..

[11] Kumar R (2015). Polarization second harmonic generation microscopy provides quantitative enhanced molecular specificity for tissue diagnostics. J. Biophotonics.

[12] Mansfield JC, Winlove CP, Moger J, Matcher SJ (2008). Collagen fiber arrangement in normal and diseased cartilage studied by polarization sensitive nonlinear microscopy. J. Biomed. Opt..

[13] Islam A, Romijn EI, Lilledahl MB, Martinez-Zubiaurre I (2017). Non-linear optical microscopy as a novel quantitative and label-free imaging modality to improve the assessment of tissue-engineered cartilage. Osteoarthr. Cartil..

[14] Kiyomatsu H (2015). Quantitative SHG imaging in osteoarthritis model mice, implying a diagnostic application. Biomed. Opt. Express.

[15] Matcher SJ (2015). What can biophotonics tell us about the 3D microstructure of articular cartilage?. Quant. Imaging Med. Surg..

[16] Finnøy A, Olstad K, Lilledahl MB (2017). Non-linear optical microscopy of cartilage canals in the distal femur of young pigs may reveal the cause of articular osteochondrosis. BMC Vet. Res..

[17] Chen X, Nadiarynkh O, Plotnikov S, Campagnola PJ (2012). Second harmonic generation microscopy for quantitative analysis of collagen fibrillar structure. Nat. Protoc..

[18] Brown C (2014). Imaging and modeling collagen architecture from the nano to micro scale. Biomed. Opt. Express.

[19] Houle MA (2015). Analysis of forward and backward Second Harmonic Generation images to probe the nanoscale structure of collagen within bone and cartilage. J. Biophotonics.

[20] Koff MF (2013). Correlation of meniscal T2* with multiphoton microscopy, and change of articular cartilage T2 in an ovine model of meniscal repair. Osteoarthr. Cartil..

[21] Zhu, X. *et al*. Quantification of collagen distributions in rat hyaline and fibro cartilages based on second harmonic generation imaging. **10024**, 1002424 (2016).

[22] Zipfel WR (2003). Live tissue intrinsic emission microscopy using multiphoton-excited native fluorescence and second harmonic generation. Proc. Natl. Acad. Sci..

[23] Zhu X (2016). Nonlinear Optical Microscopy Captures High-Resolution Images of Microstructures Within Three Types of Unlabeled Rat Cartilage. IEEE Photonics J..

[24] Ignatieva NY (2012). Two subsystems of meniscal collagen and their different thermal stabilities. Dokl. Biochem. Biophys..

[25] Donnelly E, Williams R, Farnum C (2008). The primary cilium of connective tissue cells: Imaging by multiphoton microscopy. Anat. Rec..

[26] Qu F (2015). Repair of dense connective tissues via biomaterial-mediated matrix reprogramming of the wound interface. Biomaterials.

[27] Cai H, Hao Z, Xiao L, Wan C, Tong L (2017). The collagen microstructural changes of rat menisci and tibiofemoral cartilages under the influence of mechanical loading: An *in vitro* wear test of whole joints. Technol. Heal. Care.

[28] Vesuna S, Torres R, Levene MJ (2011). Multiphoton fluorescence, second harmonic generation, and fluorescence lifetime imaging of whole cleared mouse organs. J. Biomed. Opt..

[29] Baskey Stephen J., Andreana Marco, Lanteigne Eric, Ridsdale Andrew, Stolow Albert, Schweitzer Mark E. (2019). Pre-Clinical Translation of Second Harmonic Microscopy of Meniscal and Articular Cartilage Using a Prototype Nonlinear Microendoscope. IEEE Journal of Translational Engineering in Health and Medicine.

[30] Martínez H, Brackmann C, Enejder A, Gatenholm P (2012). Mechanical stimulation of fibroblasts in micro-channeled bacterial cellulose scaffolds enhances production of oriented collagen fibers. J. Biomed. Mater. Res. - Part A.

[31] Rivard M (2011). The structural origin of second harmonic generation in fascia. Biomed. Opt. Express.

[32] Stoller P, Reiser KM, Celliers PM, Rubenchik AM (2002). Polarization-modulated second harmonic generation in collagen. Biophys. J..

[33] Erikson A, Örtegren J, Hompland T, de Lange Davies C, Lindgren M (2007). Quantification of the second-order nonlinear susceptibility of collagen I using a laser scanning microscope. J. Biomed. Opt..

[34] Ducourthial Guillaume, Affagard Jean‐Sébastien, Schmeltz Margaux, Solinas Xavier, Lopez‐Poncelas Maeva, Bonod‐Bidaud Christelle, Rubio‐Amador Ruth, Ruggiero Florence, Allain Jean‐Marc, Beaurepaire Emmanuel, Schanne‐Klein Marie‐Claire (2019). Monitoring dynamic collagen reorganization during skin stretching with fast polarization‐resolved second harmonic generation imaging. Journal of Biophotonics.

[35] Kwok J (2014). Atomic force microscopy reveals age-dependent changes in nanomechanical properties of the extracellular matrix of native human menisci: Implications for joint degeneration and osteoarthritis. *Nanomedicine*. Nanotechnology, Biol. Med..

[36] Smith SM, Shu C, Melrose J (2010). Comparative immunolocalisation of perlecan with collagen II and aggrecan in human foetal, newborn and adult ovine joint tissues demonstrates perlecan as an early developmental chondrogenic marker. Histochem. Cell Biol..

[37] Koyuncu E (2017). The morphological anatomy of the menisci of the knee joint in human fetuses. Balkan Med. J..

[38] Di Giancamillo A, Deponti D, Addis A, Domeneghini C, Peretti GM (2014). Meniscus maturation in the swine model: Changes occurring along with anterior to posterior and medial to lateral aspect during growth. J. Cell. Mol. Med..

[39] Qu, F. *et al*. Maturation State and Matrix Microstructure Regulate Interstitial Cell Migration in Dense Connective Tissues. *Sci. Rep*., **8**, (2018). 10.1038/s41598-018-21212-4 PMC5818574 29459687

[40] Légaré F, Pfeffer C, Olsen BR (2007). The Role of Backscattering in SHG Tissue Imaging. Biophys. J..

[41] Rouède D (2017). Determination of extracellular matrix collagen fibril architectures and pathological remodeling by polarization dependent second harmonic microscopy. Sci. Rep..

[42] Cheung HS (1987). Distribution of type I, II, III and v in the pepsin solubilized collagens in bovine menisci. Connect. Tissue Res..

[43] Gusachenko I, Latour G, Schanne-Klein M-C (2010). Polarization-resolved Second Harmonic microscopy in anisotropic thick tissues. Opt. Express.

[44] Romijn EI, Finnøy A, Lilledahl MB (2019). Analyzing the feasibility of discriminating between collagen types I and II using polarization-resolved second harmonic generation. J. Biophotonics.

[45] Rivard, M. Imagerie tissulaire par microscopie de seconde harmonique interférométrique - Chap. 5 Microscopie gsh interférométrique. (INRS-EMT, 2015).

[46] Notbohm H (1999). Recombinant human type II collagens with low and high levels of hydroxylysine and its glycosylated forms show marked differences in fibrillogenesis *in vitro*. J. Biol. Chem..

[47] Bai P, Hardt T, Cernadas M, Brodsky B (1992). Glycation alters collagen fibril organization. Connect. Tissue Res..

[48] Parry DAD, Flint MH, Gillard GC, Craig AS (1982). A role for glycosaminoglycans in the development of collagen fibrils. FEBS Lett..

[49] Kambic HE, McDevitt CA (2005). Spatial organization of types I and II collagen in the canine meniscus. J. Orthop. Res..

[50] Bland YS, Ashhurst DE (1996). Development and ageing of the articular cartilage of the rabbit knee joint: Distribution of the fibrillar collagens. Anat. Embryol. (Berl)..

[51] Cluzel C, Blond L, Fontaine P, Olive J, Laverty S (2013). Foetal and postnatal equine articular cartilage development: Magnetic resonance imaging and polarised light microscopy. Eur. Cells Mater..

[52] Carthage, W. Developmental horizons and measurements useful for age determination of equine embryos and fetuses. (KANSAS STATE UNIVERSITY, 1968).

[53] Fontaine P (2013). Computed tomography and magnetic resonance imaging in the study of joint development in the equine pelvic limb. Vet. J..

[54] Coluccino L (2016). Anisotropy in the viscoelastic response of knee meniscus cartilage. J. Appl. Biomater. Funct. Mater..

[55] Schindelin J (2012). Fiji: An open-source platform for biological-image analysis. Nat. Methods.

[56] Romijn EI, Finnøy A, Kumar R, Lilledahl MB (2018). Automated calibration and control for polarization-resolved second harmonic generation on commercial microscopes. PLoS One.

[57] Pinsard, M. P-SHG (forked version) GitHub. (2018). Available at: https://github.com/MaxP92/P-SHG. (Accessed: 24th August 2018)

[58] Teulon C, Tidu A, Portier F, Mosser G, Schanne-Klein M-C (2016). Probing the 3D structure of cornea-like collagen liquid crystals with polarization-resolved SHG microscopy. Opt. Express.

[59] Wasik V, Galland F, Brasselet S, Rigneault H, Réfrégier P (2016). Detection of imprecise estimations for polarization-resolved second-harmonic generation microscopy. J. Opt. Soc. Am. A.

[60] Berens, P. CircStat: A MATLAB Toolbox for Circular Statistics. *J. Stat. Softw*., **31**, (2009).

