# Supplementary material for: Maturation of the Meniscal Collagen Structure Revealed by Polarization-Resolved and Directional Second Harmonic Generation Microscopy
Source: Sci Rep. 2019 Dec 5;9:18448. doi: 10.1038/s41598-019-54942-0 (PMC6895152; doi:10.1038/s41598-019-54942-0)
Supplement: Supplementary file 1 — Supplementary information [file 41598_2019_54942_MOESM1_ESM.docx]

**MATURATION OF THE MENISCAL COLLAGEN STRUCTURE REVEALED BY POLARIZATION AND DIRECTIONAL SECOND HARMONIC GENERATION MICROSCOPY**

**Maxime Pinsard^1^, Sheila Laverty**^2,†^**, Hélène Richard**^2^**, Julia Dubuc^2^, Marie-Claire Schanne-Klein^3^ and François Légaré^1,*^**

**^1^INRS-EMT, Varennes, QC, Canada**

**^2^Faculté de médecine vétérinaire de Saint-Hyacinthe, Université de Montréal, QC, Canada**

**^3^Laboratoire d'Optique et Biosciences (LOB), Ecole polytechnique, CNRS, Inserm, Institut Polytechnique de Paris, France**

**E-mails :^†^sheila.laverty@umontreal.ca ;** [**^*^legare@emt.inrs.ca**](mailto:*legare@emt.inrs.ca)

**SUPPLEMENTARY MATERIAL**

Table S1: Different meniscus samples used in the study: N=6 for adults and N=5 for foetuses.

| Type | Number | Side | Age (Years) | Orientation distribution |
| --- | --- | --- | --- | --- |
| Foetus | F1 | Right, lateral | -0.44 | homogeneous |
|  | F2 | Right, medial | -0.49 | complex, random |
|  | F3 | Right, medial | -0.6 | homogeneous |
|  | F4 | Right, medial | -0.66 | complex, random |
|  | F5 | Right, lateral | -0.9 | complex, random |
| Adult | A1 | Left, medial | 4 | clusters |
|  | A2 | Right, medial | 8 | clusters |
|  | A3 | Right, medial | 9 | clusters |
|  | A4 | Right, medial | 10 | clusters |
|  | A5 | Right, medial | 11 | clusters |
|  | A6 | Right, medial | 14 | clusters |


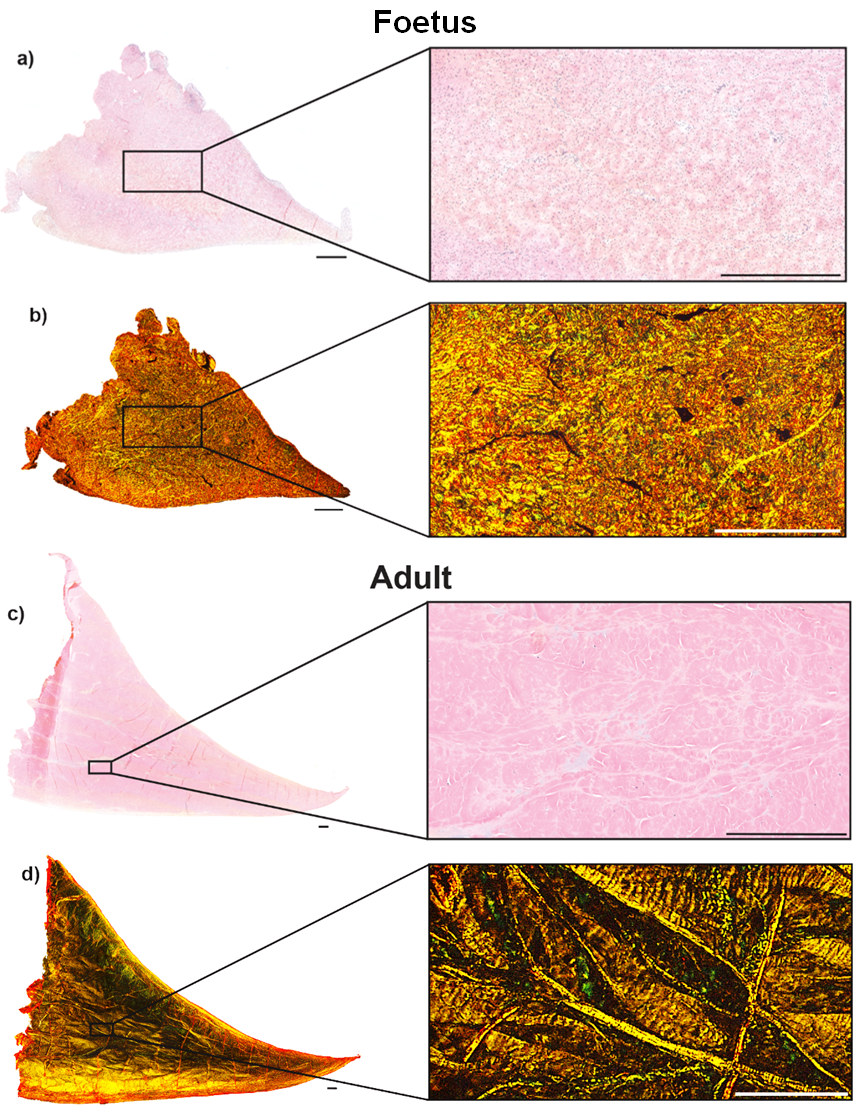


Figure S1: Whole meniscus sections (left) of foetus F2 (a & b) and adult A4 (b & c), and a zoom in the zone imaged using SHG microscopy (right). (a & c) HEPS staining and (b & d) Picro-sirius staining (revealing the collagen fibrils), showing that differences in the collagen organization are poorly evidenced using these staining methods. Scale-bar: 500μm.


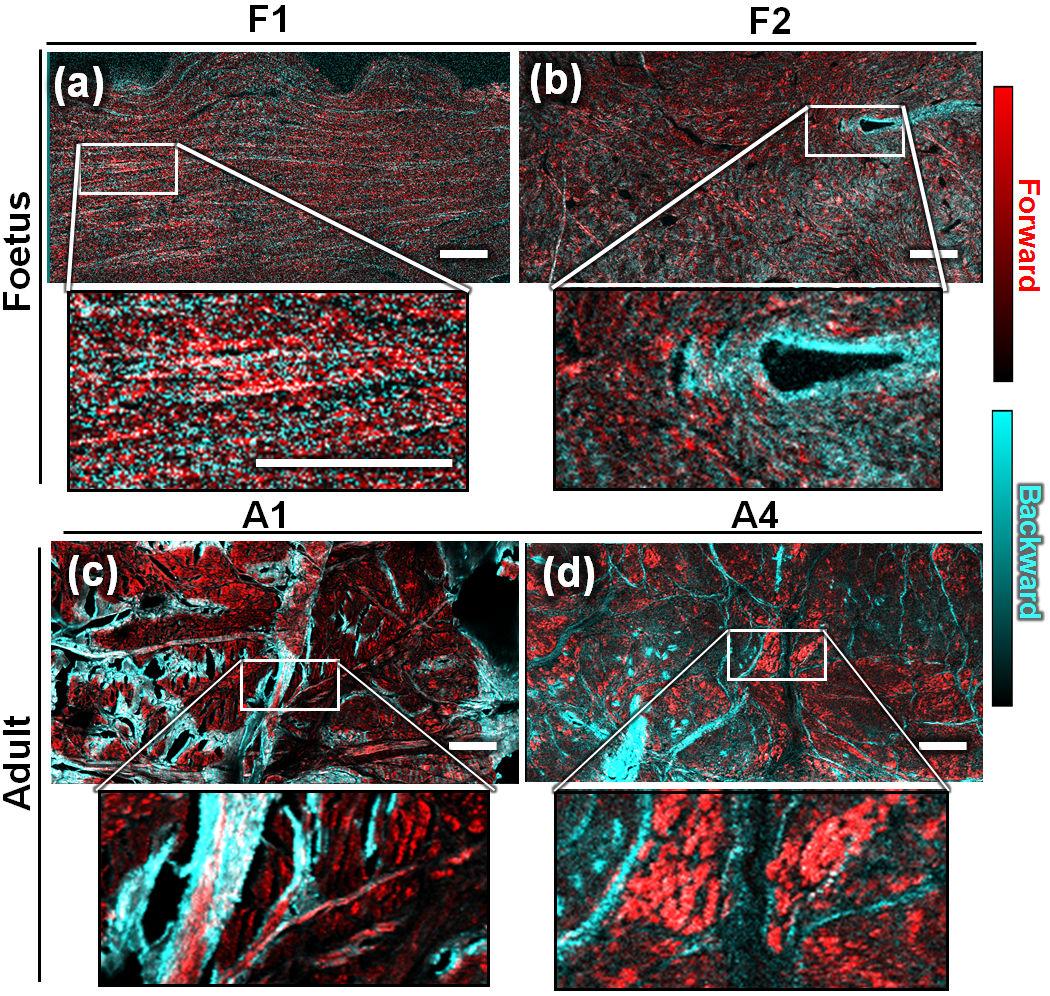


Figure S2: Merger of forward SHG (in red), and backward SHG (in cyan) images of Figure 1 (foetus F1 and F2) and Figure 2 (adults A1 and A4). The signal of the backward channel is enhanced 8× for (a), 4.1× for (b), 6.9× for (c) and 32× for (d) to be comparable to the one of the forward channel. Scale-bars: 200 μm.


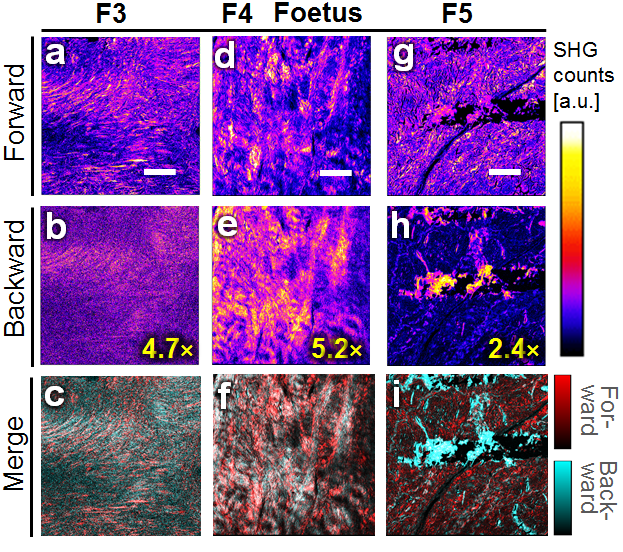


Figure S3: Forward SHG (top), backward SHG (middle) and merged (bottom) images of three menisci samples of equine foetuses (F3 (a, b, c), F4 (d, e, f), F5 (g, h, i)).

The meniscus F3 shows a homogeneous tissue (orientation rather horizontal), especially in backward direction, whereas the menisci F4 and F5 show more randomly oriented collagen fibrils. The menisci F3 and F4 show similar images in forward and backward directions. The images of the same samples (a-b, d-e or g-h) are displayed using the same look-up table, but the backward images have been multiplied by a factor indicated in yellow because less signal is physically detected in this direction compared to forward. In the merged image, the forward channel is in red, and the backward in cyan (and use the same multiplicative factors as in b, e, h). Scale-bars: 200μm.


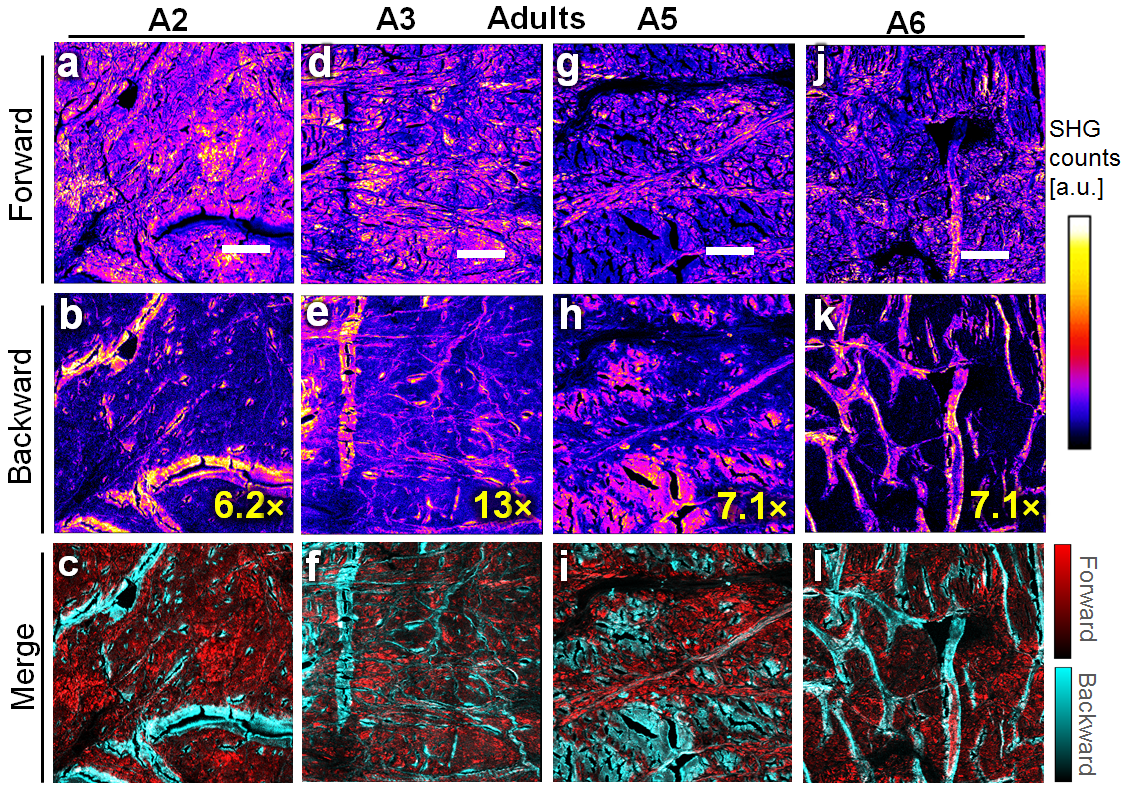


Figure S4: Forward SHG (top), backward SHG (middle) and merged images (bottom) of four menisci samples of equine adults (A2 (a, b, c), A3 (d, e, f), A5 (g, h, i) and A6 (j, k, l)).

All these menisci show different patterns in forward and backward directions: the backward image better highlights the thick fibers. The images of the same samples (a-b, d-e or g-h) are displayed using the same look-up table, but the backward images have been multiplied by a factor indicated in yellow because less signal is physically detected in this direction compared to forward. In the merged image, the forward channel is in red, and the backward in cyan (and use the same multiplicative factors as in b, e, h, k). Scale-bars: 200μm.

**
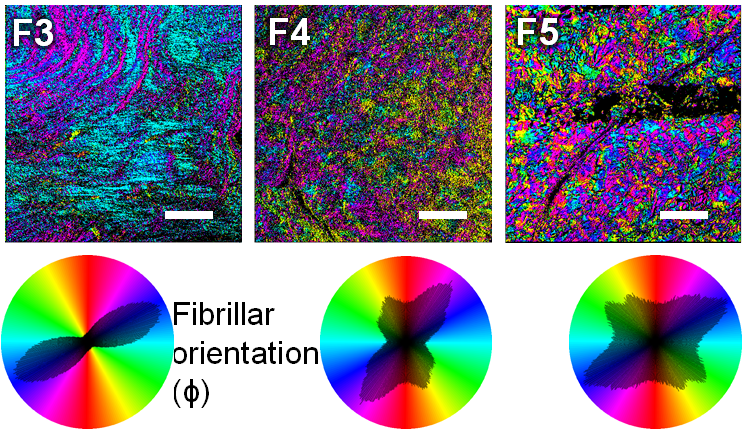
**

Figure S5: Collagen fibrils orientation in the imaging plane (ϕ) of equine foetuses (F3, F4 , F5) obtained by P-SHG (complement of Figure 4).

Since the values of ϕ are circular, the histograms are represented as polar plots on the look-up-table wheels. Foetuses show either a homogeneous orientation (left) or a random one, almost equally scattered over all the angles (middle F4 and right F5). Scale-bars: 200μm.


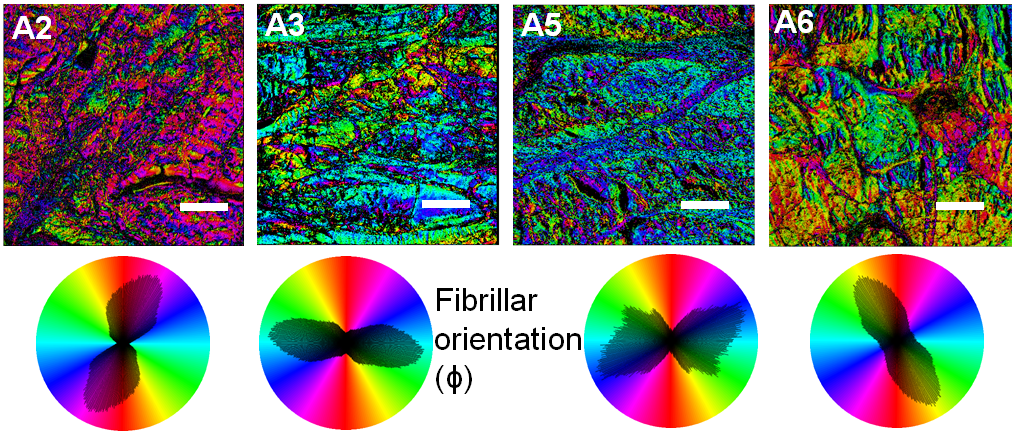


Figure S6: Collagen fibrils orientation in the imaging plane (ϕ) obtained by P-SHG of equine adults (A2, A3, A5 and A6, complement of Figure 5). Since the values of ϕ are circular, the histograms are represented as polar plots on the look-up-table wheels. Adult menisci show a well-organized structure, with some thick fibers clearly identified from the inter-fiber areas. Scale-bars: 200μm.


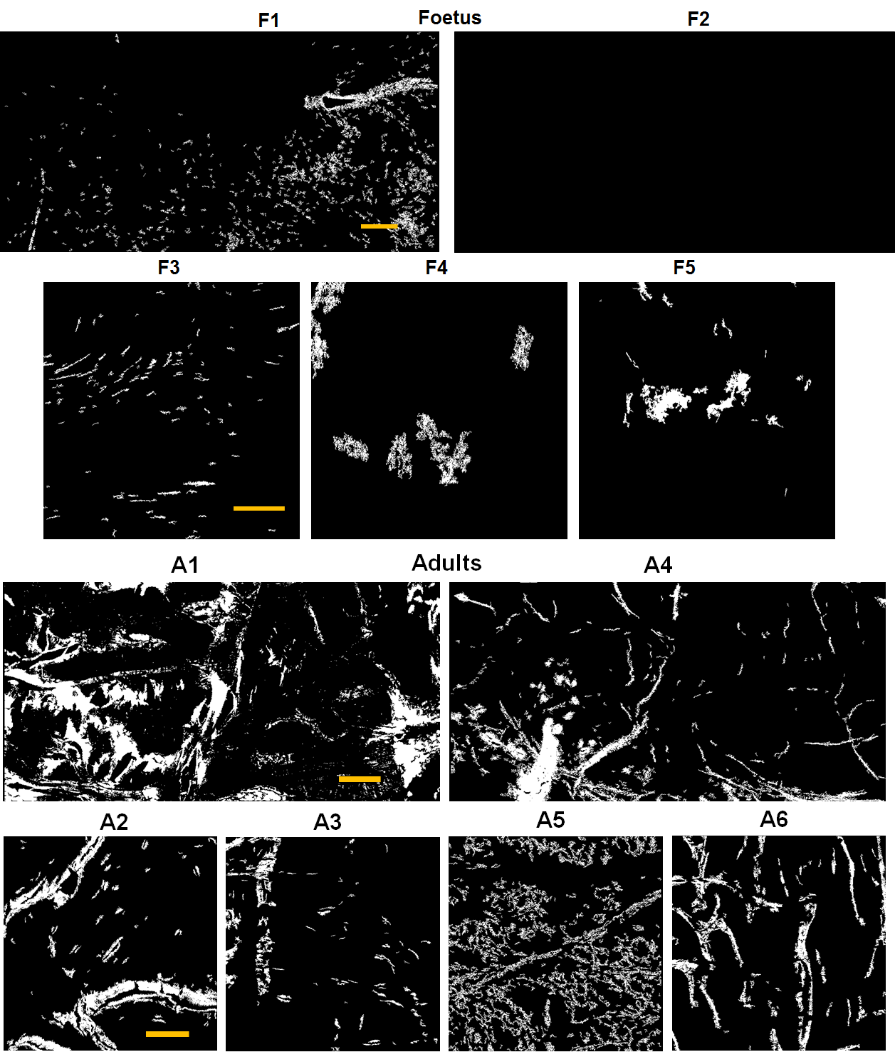


Figure S7: Masks obtained for all the samples, using an intensity threshold (ImageJ "Yen white") and a noise filtering, to differentiate the inter-fibers areas (in black) from the thick structures (in white). These masks are used for the measures of Figure 6. Scale-bars 200μm.
